# A High-Performing Sulfur-Tolerant and Redox-Stable Layered Perovskite Anode for Direct Hydrocarbon Solid Oxide Fuel Cells

**DOI:** 10.1038/srep18129

**Published:** 2015-12-09

**Authors:** Hanping Ding, Zetian Tao, Shun Liu, Jiujun Zhang

**Affiliations:** 1 School of Petroleum Engineering, Xi’an Shiyou University, Xi’an 710065, China; 2 Key Laboratory for Advanced Technology in Environmental Protection of Jiangsu, Province, Yancheng Institute of College, Yancheng, Jiangsu Province, China; 3 Colorado Fuel Cell Center, Department of Mechanical Engineering, Colorado School of Mines, Golden CO 80401, USA; 4 Energy, Mining & Environment, National Research Council of Canada, Vancouver, BC V6T 1W5, Canada

## Abstract

Development of alternative ceramic oxide anode materials is a key step for direct hydrocarbon solid oxide fuel cells (SOFCs). Several lanthanide based layered perovskite-structured oxides demonstrate outstanding oxygen diffusion rate, favorable electronic conductivity, and good oxygen surface exchange kinetics, owing to A-site ordered structure in which lanthanide and alkali-earth ions occupy alternate (001) layers and oxygen vacancies are mainly located in [LnO_*x*_] planes. Here we report a nickel-free cation deficient layered perovskite, (PrBa)_0.95_(Fe_0.9_Mo_0.1_)_2_O_5 + δ_ (PBFM), for SOFC anode, and this anode shows an outstanding performance with high resistance against both carbon build-up and sulfur poisoning in hydrocarbon fuels. At 800 °C, the layered PBFM showed high electrical conductivity of 59.2 S cm^−1^ in 5% H_2_ and peak power densities of 1.72 and 0.54 W cm^−2^ using H_2_ and CH_4_ as fuel, respectively. The cell exhibits a very stable performance under a constant current load of 1.0 A cm^−2^. To our best knowledge, this is the highest performance of ceramic anodes operated in methane. In addition, the anode is structurally stable at various fuel and temperature conditions, suggesting that it is a feasible material candidate for high-performing SOFC anode.

Solid oxide fuel cell (SOFC), an electrochemical device that can directly convert chemical energy to electricity, has become a feasible technology for energy-supply due to its high-energy conversion efficiency, wide application range and fuel flexibility^1^,^2^,^3^,^4^,^5^. Normally, a SOFC operated at high temperatures can essentially convert any fuel, such as hydrogen, alcohols, hydrocarbons, or even carbon into electricity^6^. Particularly, when using hydrocarbon fuels such as natural gas to produce electricity, SOFC has been recognized to be the most promising device with high conversion and energy efficiencies. As identified, the anode plays a critical role in SOFC performance and durability, particularly when a hydrocarbon is used as the fuel. Conventionally, Ni-based composites which give high activity for pure H_2_ oxidation and also good current collection are most commonly used as the anode materials^7^. However, they also exhibit some disadvantages such as low tolerance to coking (carbon deposition) unless a large amount of steam is added to reform the fuel, vulnerability to sulfur intrinsically existing in natural fuels due to the formation of NiS compound^8^, and nickel coarsening as well as poor volume stability upon redox cycling. To overcome these challenges in maximizing the full advantage of the intrinsic fuel flexibility of SOFC, early efforts have been made to develop alternative anode materials. For example, replacing the traditional anode with a Cu-ceria-YSZ composite one has been reported to reduce anode carbon deposition to make the SOFC operation in a range of dry hydrocarbons^9^,^10^. However, it was found that the inactive Cu particles with poor catalytic activity could limit the cell performance and also suffer coarsening over time owing to the low melting point. In order to obtain a high-performing coking-resistant anode, Zhan *et al.* introduced a thin catalytic layer of Ru-CeO_2_ that is placed against the anode side, allowing internal reforming of iso-octane without coking and yielding stable power density of 0.6 W cm^−2^ at 770 °C^11^. This innovative approach showed promising but expensive.

Several oxides with a perovskite structure have also been explored as the anode materials, which are mixed ionic-electronic conductors in the reducing condition and catalytically more active than ceria for oxidation of hydrocarbon fuels. Perovskites could readily accept oxygen vacancies and contain transition-metal cations in the octahedral sites due to the high tolerance factor against crystal distortion. Based on these beneficial factors of perovskites, several oxides, such as La_0.75_Sr_0.25_Cr_0.5_Mn_0.5_O_3_ (LSCM)^12^, La_0.33_Sr_0.67_Ti_1−x_M_x_O_3_ (LSTO, M = Fe^n+^, Mn^n+^, Sc)^13^, Sr_2_MMoO_6_ (SMMO, M = Mg, Fe, Co)^8^,^14^,^15^ and Pr_0.8_Sr_1.2_(Co,Fe)_0.8_Nb_0.2_O_4_ (K-PSCFN)^16^, have been investigated as the potential anode materials. These conductive anode materials having high resistance against both coking and sulfur poisoning could show some stability in reducing condition. However, these anodes showed some limitations, such as insufficient electrical conductivity and low catalytic activity when compared to those of the conventional Ni-YSZ anode. For instance, without a Pd or Ni catalyst, pure LSTO or LSCM anode could not provide reasonable performance in H_2_ below 900 °C and its catalytic activity toward CH_4_ oxidation seemed insufficient^17^,^18^. It was also observed that the catalytic pathways for reforming methane during the cell operation could be blocked by the residue (SrCO_3_ and SrMoO_4_) on the surface of SMMO anode^19^.

In the effort to develop high-performing SOFC anodes, we have synthesized a highly redox-stable ceramic oxide with an A-site deficient layered perovskite structure, i.e. (PrBa)_0.95_(Fe_0.9_Mo_0.1_)_2_O_5+δ_ (PBFM) in this work, and when this material is used for the anode, an outstanding electrochemical activity toward fuel oxidation in a direct hydrocarbon fueled SOFC is achieved. Our strategy in the selection of this material is based on the following observations: (1) Perovskites with high tolerance against crystal structure distortion could allow to tailor the material’s chemical stability and also the electrical/catalytic/mechanical properties through doping strategy; (2) Fe-rich perovskite containing mixed-valence Fe^2+^/Fe^3+^ redox couple could provide high electronic conductivity even though these redox ions only partially occupy the sub-lattice; (3) Layered perovskite structure could give a high electrical conductivity and the ordered A-cations localizing oxygen vacancies within the rare earth layers, which could make a contribution to the fast oxygen surface exchange/bulk diffusion and catalytic activity towards both hydrogen and hydrocarbon oxidation processes; and (4) Our experiments showed that this PBFM was highly stable upon partial removal of lattice oxygen, and that the use of sixfold-coordinated Mo(VI)/Mo(V) couple at B site could stabilize the material with stronger chemical bond against crude anodic conditions^8^.

## Results

### Characterization of PBFM anode

**Figure 1:**
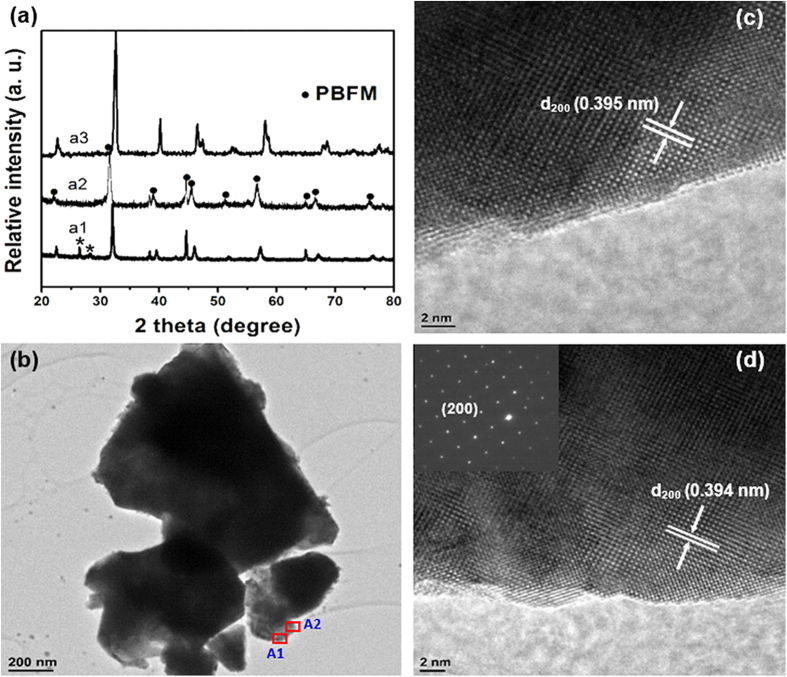
Material analysis. (**a**) XRD of PBFM, obtained after a1) 1000 °C calcination for 3 hours in air (asterisk correspond to impurity phase of BaMoO_4_) and a2) 900 °C calcination for 5 hours in 5% H_2_; and a3) PrBaFe_2_O_5 +_ δ before B-site doping. (**b**) Bright-field TEM image of powder morphology. (**c**) High-resolution TEM lattice fringe image of boxed area A1. (**d**) Area A2 and corresponding SAED pattern (insert).

Figure 1 shows a single-phase layered perovskite structure of PBFM obtained by firing in air at 1000 °C and subsequently in 5% H_2_ environment at 900 °C (Fig. 1a, Trace a1 and a2). As observed, a pure phased PBFM could not be obtained if the sample was fired only in air, during which a large portion of BaMoO_4_ as a second phase was produced. In this case, a further baking treatment (the sample was calcined at 900 °C in 5% H_2_) in a reducing atmosphere was found to be needed in order to compromise the new charge-neutrality balance induced by the incorporation of larger Mo^6+^ ions with higher valence than Fe^3+^. After the treatment, a thermogravimetri analysis (TGA) showed that the substantial lattice oxygen was lost in the phase-formation process (Supplementary Fig. 1).

The crystal structure of this PBFM material presents two structural features: (1) Pr^3+^ and Ba^2+^ ions do not form a solid solution at A-site but are ordered in alternating (001) layers. A significant difference in size between the large Ba cation and the small Pr cation results in the formation of alternating [PrO] and [BaO] layers along the c-axis with a stacking sequence of … |BaO|FeO_2_|PrO_*x*_|FeO_2_| ….; and, (2) oxygen vacancies are mainly located at the [PrO_*x*_] plane with a great tendency to form ordered patterns under reducing conditions (Supplementary Fig. 2). As a result, a coexistence of Fe ions in octahedral and pyramidal environments in an ordered manner can be observed^20^,^21^,^22^. It is believed that the oxygen-ion diffusion in such a doped perovskite can be enhanced by orders of magnitude if a simple cubic crystal can transform into a layered compound with ordered Pr and Ba ions. The layered structure could reduce the oxygen bonding strength and provides disorder-free channels for ion motion, resulting in lower activation energy and rapid oxygen surface exchange coefficient.

As shown in the bright-field TEM image (Fig. 1b), the as-prepared PBFM powder has a smooth surface morphology. In Fig. 1c,d, the lattice-resolved high resolution TEM (HRTEM) images of the grain edge show the presence of highly crystalline nature, which corresponds to the (200) crystal plane of the double perovskite structure with a lattice inter-planar spacing of *d*_200_ = 0.394 nm. The selected area electron diffraction (SAED) pattern of boxed area (A2 in Fig. 1b) confirms the long-range order crystal structure. The element contents in the nanoparticles as determined by an energy dispersive X-ray (EDX) analysis equipped in TEM showed the existence of Pr, Ba, Fe and Mo, where Cu and C were from the substrate of sample stage (Supplementary Fig. 3). The atomic ratio of Pr, Ba, Fe and Mo was determined to be about 5 : 5 : 9 : 1, which was fairly close to the stoichiometric composition. Because the small fraction of 0.1 for Mo ions at B site, the coexistence of A and B site cation ordering might not be observable, even if the phase exists as AA'BB'O_6_-type structure. Furthermore, no chemical reaction can be found when firing a mixture of PBFM and LSGM at 1000 °C in air for 100 hours, indicating a good chemical compatibility (Supplementary Fig. 4). The thermal expansion coefficient was measured to be 11.96 × 10^-6^ 1/K, which is very close to that of LSGM, and other commonly used electrolytes (Supplementary Fig. 5). In addition, PBFM with A-site cation ordering structure is found to be very stable under fuel conditions. The layered perovskite structure can be retained when it is fired in 5% H_2_/95% Ar at even as high as 1000 °C for 200 hours (Supplementary Fig. 6) while the shift of diffraction peaks can be found.

### Electrical and catalytic properties in various conditions

**Figure 2:**
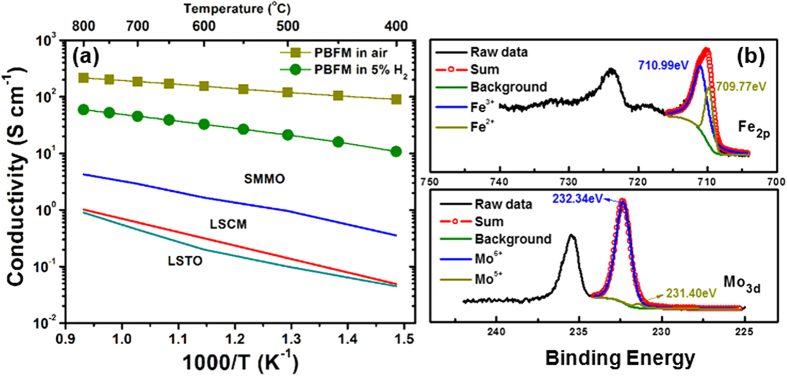
Electrical conductivity and XPS study. **(a)** Temperature dependence of electrical conductivities of SOFC anode PBFM material, measured in air and 5% H_2_, compared to other three anode materials (LSCM^12^, LSTO^13^ and SMMO^14^), measured in the same condition. **(b)** Fe 2p and Mo 3d XPS spectra of layered PBFM sample at room temperature.

For an oxide-based SOFC anode, in order to obtain a comparable to or better performance than that of conventional Ni/YSZ cermet anode, its electrical conductivity should be sufficient for improved catalytic activity and current collection efficiency. Figure 2a shows the electrical conductivity of the PBFM as a function of temperature in air and wet 5% H_2_, respectively. It can be clearly seen that PBFM developed in this work has much high conductivities than those of three other ceramic oxide anodes (LSCM^12^, LSTO^13^ and SMMO^14^), suggesting that PBFM should be a high-performing anode material in terms of electrical conductivity. This new PBFM material with A-site cation ordered structure could retain high electrical conductivities of 217 S cm^−1^ in air and 59.2 S cm^−1^ in 5% H_2_ at 800 °C, respectively. As shown in Fig. 2b, the X-ray photoelectron spectroscopy (XPS) results indicate that the major oxidation states of Fe and Mo in PBFM are + 3 and + 6, respectively. The atomic ratios of Fe^3+^/Fe^2+^ and Mo^6+^/Mo^5+^ couples are determined to be 2.71 : 1 and 28.76 : 1, respectively, indicating the nature of mixed electronic and ionic conductor. In air, hole conduction should be predominant in PBFM with reasonable oxygen ion conductivity induced by the substantial oxygen vacancy concentration present in [PrO_*x*_] crystal planes. After reduction in 5% H_2_ for 20 hours at 800 °C, the conductivity of PBFM was found to decrease due to the lowered mean Fe valence, but its oxide ionic conductivity was increased due to more available oxygen vacancies, which was consistent with the TGA result that the substantial lattice oxygen is lost above 400 °C in 5% H_2_, as shown in Supplementary Fig. 1.

**Figure 3:**
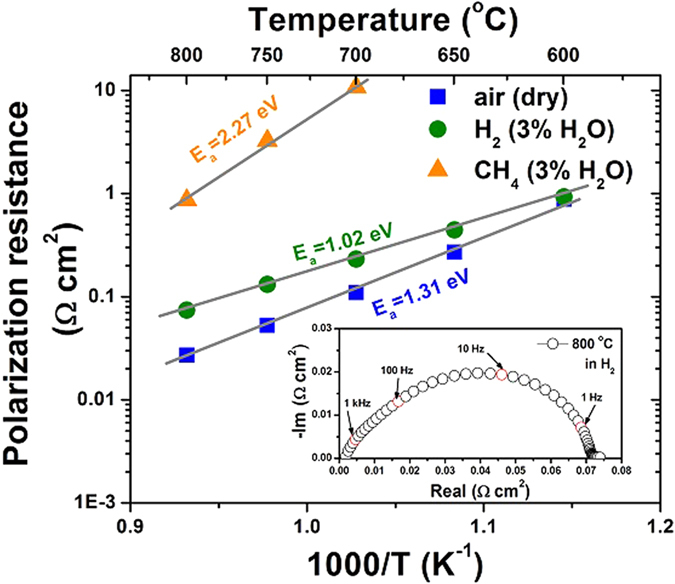
Area specific resistance (ASR) of the PBFM anode under different atmospheres: dry air, humid H_2_ and CH_4_ (~3% H_2_O), as a function of temperature. The insert is a typical impedance spectrum, as obtained from symmetric cell of PBFM|LSGM |PBFM at 800 °C in H_2_. The ohmic resistance from LSGM electrolyte has been subtracted for clear comparison of electrode polarization.

In order to evaluate the electrode performance in different gas conditions, a symmetric half cell using PBFM material as both working and counter electrodes on a LSGM (La_0.9_Sr_0.1_Ga_0.8_Mg_0.2_O_3_) electrolyte was prepared, with an Au paste applied as current collector on the both sides. As shown in Fig. 3, the PBFM electrode polarization resistances were measured under open circuit conditions in air, wet hydrogen and wet methane atmospheres and different temperatures. It can be seen that the electrode polarization resistances in air are 0.027 Ω cm^2^, 0.11 Ω cm^2^, and 0.88 Ω cm^2^ at 800 °C, 700 °C and 600 °C, respectively, which are comparable to commonly used cathode materials such as La_0.8_Sr_0.2_MnO_3_ (4.2 Ω cm^2^ at 700 °C)^23^, and La_x_Sr_1−x_Co_y_Fe_1-y_O_3-δ_ (0.34 Ω cm^2^ at 700 °C)^24^, indicating the excellent catalytic activity of PBFM for oxygen reduction reaction.

As shown in Fig 3, the electrode polarization resistances of PBFM anode in H_2_ are 0.074, 0.132 and 0.231 Ω cm^2^ at 800 °C, 750 °C and 700 °C, respectively, which are lower than previously developed oxide anodes. For instance, the LSCM anode developed by Tao *et al.*^12^ exhibited a polarization resistance of 0.26 Ω cm^2^ in wet H_2_ at 900 °C. For a recently well-developed Sr_2_Fe_1.5_Mo_0.5_O_6_ anode, the lowest polarization resistance of 0.21 Ω cm^2^ was reported at 800 °C in H_2_^15^. Furthermore, this PBFM anode can even give very promising performance when operation temperature is decreased: 0.44 Ω cm^2^ at 650 °C and 0.93 Ω cm^2^ at 600 °C, respectively. As calculated, the activation energy of PBFM in H_2_ was 1.02 eV, lower than 1.31 eV in air, indicating an advantage if this material is used for SOFC anode. When the gas condition is switched to methane (~3% H_2_O), the polarization resistance is consequently raised up to 0.86 Ω cm^2^ at 800 °C, 3.25 Ω cm^2^ at 750 °C and 10.76 Ω cm^2^ at 700 °C, respectively.

### Power output and durability of fuel cells in H_2_, CH_4_ and H_2_S-containing H_2_

**Figure 4:**
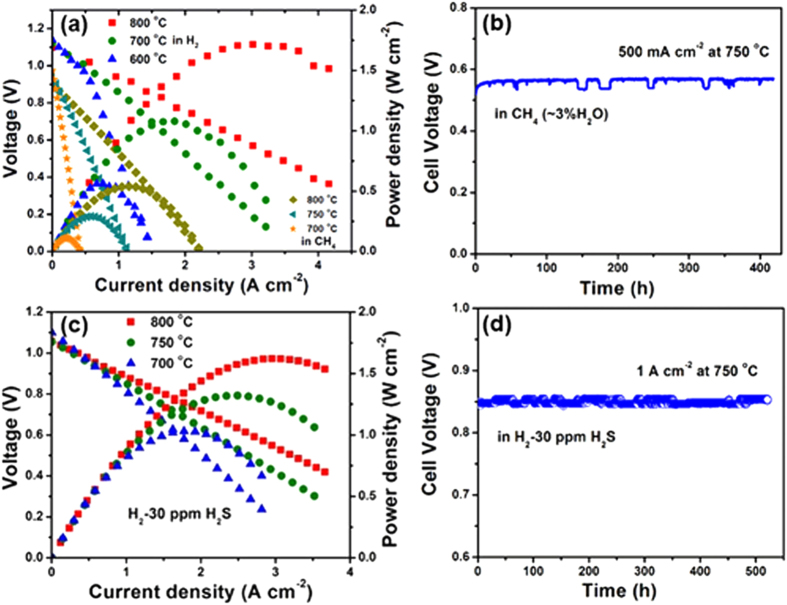
Electrochemical performances obtained from a LSGM (~200 μm)-based SOFC with layered PBFM anode and PrBaCo_2_O_5+δ_ cathode. **(a)** Cell voltage and power density as a function of current density at different temperatures in H_2_ and CH_4_. The 3% H_2_O humidified H_2_, CH_4_ and 30 ppm H_2_S contained H_2_ while static ambient air was used as oxidant. **(b)** Long-term stability test in CH_4_ (~3% H_2_O) under a constant current load of 0.5 A cm^−2^ at 750 °C. **(c)** Performance obtained from 700 to 800 °C in H_2_-30 ppm H_2_S. **(d)** Long-term stability test at 750 °C under a constant current load of 1 A cm^−2^ in H_2_-30 ppm H_2_S.

Figure 4a shows the electrochemical performance of a LSGM electrolyte-supported SOFC with the configuration of PBFM|LSGM|PBCO (PrBaCo_2_O_5+δ_), tested using various humidified (~3% H_2_O) fuels (H_2_ and CH_4_) and ambient air as oxidant. It can be seen that the open circuit voltage (OCV) for wet H_2_ is close to the theoretical value calculated by the Nernst equation, 1.12 V at 800 °C, 1.14 V at 700 °C and 1.16 V at 600 °C, respectively. The maximum power density (P_max_) can reach up to 1.72, 1.05 and 0.56 W cm^−2^ at 800, 700 and 600 °C, respectively, and the cell exhibits a very stable performance under a constant current load of 1.0 A cm^−2^ at 700 °C for 450 hours without any degradation (Supplementary Fig. 7). The OCVs of the cell using wet (3%H_2_O) methane as fuel can reach to 0.9 V at 800 °C, 0.94 V at 750 °C and 0.97 V at 700 °C, respectively. PBFM anode can show a high maximum power density of 0.54 W cm^−2^ at 800 °C. To our best knowledge, this is the highest performance of ceramic anodes operated in methane. The impedance spectra measured under open circuit condition with wet H_2_ and CH_4_ as fuels are shown in Supplementary Fig. 8. It can be seen that the overall electrode polarization resistance is as small as 0.057 Ω cm^2^ in H_2_ and 0.255 Ω cm^2^ in CH_4_ at 800 °C, respectively, and the long-term durability test shows no obvious degradation when the cell was discharged at 0.5 A cm^−2^ and 750 °C for 420 hours (Fig. 4b). The electrochemical performance of PBFM anode can compare favorably to those of previously reported high-performance of direct hydrocarbon fueled SOFCs with ceramic anodes (Supplementary Table 1). Yoo *et al.*^25^ reported a maximum power density of ~0.63 W cm^−2^ in H_2_ at 800 °C for a LSGM (~250 μm) electrolyte supported SOFC with Ni-impregnated La_0.2_Sr_0.8_Ti_0.98_Co_0.02_O_3_-GDC composite anode. With La_0.3_Sr_0.7_TiO_3_ anode infiltrated by Pd (0.5 wt%) and CeO_2_ (5 wt%), the maximum power density was increased to 0.78 W cm^−2^ at 800 °C^26^. Liu *et al.*^15^ reported a cell performance of 0.84 W cm^−2^ in H_2_ and 0.23 W cm^−2^ in CH_4_ at 900 °C for Sr_2_Fe_1.5_Mo_0.5_O_6_ anode, and recently, Yang *et al.*^16^ reported a very promising cell performance of 0.96 W cm^−2^ in H_2_ and 0.6 W cm^−2^ in CH_4_ at 850 °C for a K_2_NiF_4_-type K-PSCFN-CFA anode with Co-Fe alloy, which were still lower than the performance in this work. It should be noted that the thin electrolyte LSGM with thickness of only 200 μm and catalytically active cathode PBCO also contributed to the high performance.

In the performance optimization, it was found that the electrode polarization resistances could be further improved by optimizing the microstructure of PBFM anode or PBCO cathode. In the experiments, a single cell with nanostructured anode microstructure (Supplementary Fig. 9) of the prime backbones infiltrated by the PBFM solution precursors with stoichiometric amounts was fabricated. The as-prepared cell shows a further enhanced performance, for example, the power densities of 2.3, 1.5 and 0.8 W cm^−2^ at 800, 700 and 600 °C in H_2_ can be achieved, respectively (Supplementary Fig. 10). Furthermore, power densities of 0.76 W cm^−2^ in CH_4_ and 2.02 W cm^−2^ in H_2_-30 ppm H_2_S at 800 °C can also be obtained. These high-performing results demonstrate the great potential of PBFM to be applied as oxide anode in high-performance SOFC operated in various fuels.

In order to determine the sulfur tolerance of PBFM anode, the cell performance in H_2_ containing H_2_S contaminant (H_2_ + ppm H_2_S) at different concentrations were tested. As shown in Fig. 4c, the maximum power densities of the cell are 1.62, 1.32 and 1.03 W cm^−2^ at 800, 750 and 700 °C in H_2_ containing 30 ppm H_2_S (Fig. 4c). In the experiments, when the fuel is switched to H_2_ + 60 ppm H_2_S and then H_2_ + 100 ppm H_2_S, the values of P_max_ can still maintain at 1.25 and 1.18 W cm^−2^, respectively, as seen in Supplementary Fig. 11. The cell under a constant current load of 1.0 A cm^−2^ exhibits a very stable durability in H_2_ + 30 ppm H_2_S at 750 °C, with a very small voltage drop in 520 hours (Fig. 4d). To examine the performance response to the fuel change, the cell with the PBFM anode was also examined when the fuel was switched between H_2_ and CH_4_. As shown in Supplementary Fig. 12, a constant current density of 0.8 A cm^−2^ is applied at 750 °C with monitoring the cell voltage change. It can be seen that a sharp decrease in cell voltage from 0.89 V to 0.3 V can be observed after the fuel is switched from wet H_2_ to CH_4_ due to the lower catalytic activity of PBFM anode for CH_4_. The cell voltage can be immediately recovered to a slightly higher value of 0.9 V after the fuel gas is switched from CH_4_ back to wet H_2_. Similar behavior can also be observed when the fuel gas is switched between wet H_2_ and H_2_ + 100 ppm H_2_S. These results demonstrate that the PBFM anode has a fast response and recovery ability to the fuel change and contamination.

### Redox stability of PBFM anode

**Figure 5:**
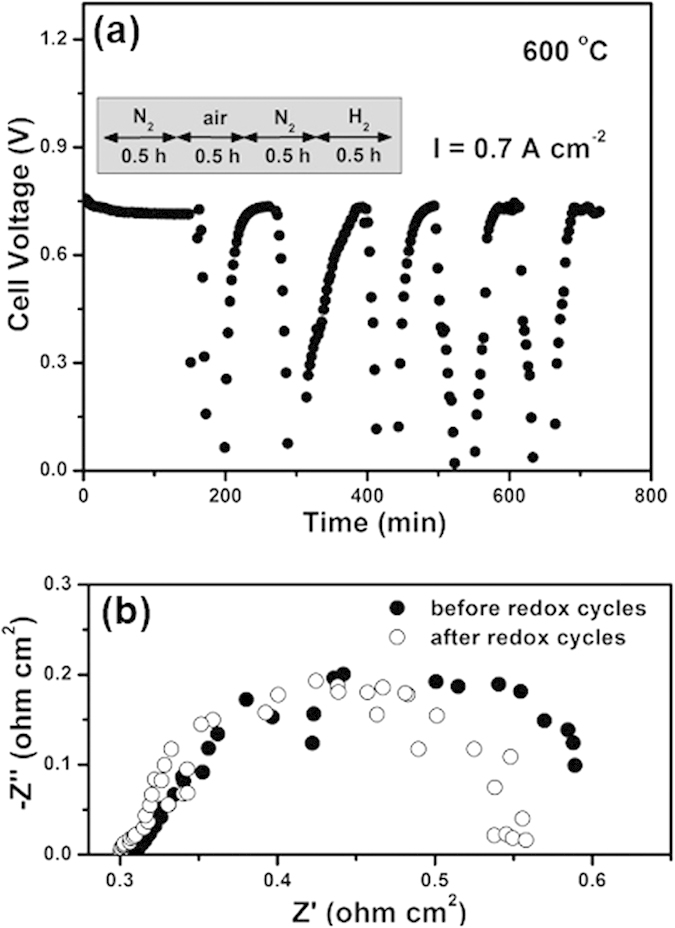
Redox stability of the PBFM|LSGM|PBCO fuel cell at 600 °C. (**a**) In a typical redox cycling test, the fuel gas is switched between air and H_2_, with N_2_ to purge the anode chamber. Under a constant current density of 0.7 A cm^−2^, the instant response of cell voltage is recorded all times. **(b)** Impedance spectra are measured under open-circuit condition before and after the redox cycles. In a typical redox cycle, H_2_ flow is stopped to feed the anode which is then purged with N_2_ for 0.5 hour, air is then flowed for 0.5 hour.

Normally, the poor redox tolerance of nickel cermet anode can preclude many medium- and small-scale applications, caused by a volume instability. Therefore, redox cycling stability is another critical aspect in evaluating an anode material’s performance for SOFCs, which is tested by switching the fuel gas between H_2_ and air at the anode side. As shown in Fig. 5a, the cell is initially operated under a constant current load of 0.7 A cm^−2^ in H_2_ at 600 °C for 3 hours to obtain a stable cell performance as baseline. Subsequently, the PBFM anode is subjected to the first redox cycle by sweeping air for 0.5 hour and then back to H_2_ for other 0.5 hour. It can be seen that the current density is firstly dropped to 0 V upon oxidation and then quickly recovered to initial value and stabilized. After five redox cycles, there is no any degradation on cell voltage. In addition, the impedance spectra of the fuel cell are also measured before and after the redox cycles, as shown in Fig. 5b. It can be seen that the major impact of redox cycling is on the value of electrode polarization resistance (with a slight decrease) rather than on the ohmic resistance, demonstrating that this anode material has a remarkable redox stability. As shown in Supplementary Fig. 13, the microstructure of post-test cells is also examined by scanning electron microscopy, showing a good adhesion between two ceramic layers.

## Discussion

Regarding the roles of Fe and Mo in the perovskite materials, Goodenough and Huang^27^ and Lindén *et al.*^28^ indicated that it might not be possible to reduce all the Fe^3+^ ions completely to Fe^2+^ in recently developed anode material of Sr_2_FeMoO_6_ because the Mo^5+^/Mo^6+^ redox band overlaps with the Fe^2+^/Fe^3+^ couple, therefore protecting the Fe^3+^ in the perovskite structure from being fully reduced. This is consistent with our results that the crystal structure of PBFM could show a high resistance against reducing condition. It is worthwhile to note that the strategy of material selection in this work is distinguished from conventional exploration of oxide anodes for SOFCs. The most popularly used LSCM and LSTO anodes are developed on the basis of obtaining the stable crystal in both reducing and oxidizing atmospheres due to highly tolerant TiO_6_ or CrO_6_ octahedra with preference of six-fold coordination in its chemistry^29^. For example, LSCM is originated from the ceramic interconnect material of LaCrO_3_, which is stable and conductive in both fuel and air conditions. To make them workable for SOFC anodes, doping strategy is generally adopted to gain reasonable ionic and electronic conductivity for electrochemical reactions. The replacement of a stable B-site ion by another active element, for example, Mn partially replacing Cr, Co partially replacing Ti, would create a new material that compromises between stability and activity while the two elements act in a complementary fashion. On the contrary, the active material might be stabilized with partially replacement of stable element while the structural, electrical and catalytic properties may be maintained to the largest extent. In this regard, conventional multivalent elements (Mn, Fe, Ni, etc.) could not only serve to compensate the creation of oxygen vacancy and function as charge carriers for electrons or holes, but also give certain stability in weak reducing condition^30^. Some typical examples include the La_0.7_Sr_0.3_FeO_3-δ_^31^ and Ba_0.95_La_0.05_FeO_3-δ_^32^ for oxygen permeation and membrane conversion. In order to stabilize the structure of these active perovskites, some elements such as Ti, Cr or Mo may be used to partially dope B-site. For layered perovskite PrBaFe_2_O_5+δ_, when 10% B-site cation is replaced by Mo, the crystal structure could become very resistant against H_2_ from extrusion of metal elements, and also contain necessarily adequate charge carriers for electrochemical reactions. For PBFM developed in this work, the XPS analysis discussed above can validate the coexistence of Fe^3+^/Fe^2+^ and Mo^6+^/Mo^5+^ couples with content ratios of 2.71 : 1 and 28.76 : 1, respectively, which are believe to be able to make a contribution to both conductivity and stability (Fig. 2). In 5% H_2_, the retained electrical conductivity demonstrates the availability of charge carriers in a broad range of oxygen partial pressure, which is comparable to other Ni-free oxide anodes, such as La_0.3_Sr_0.7_TiO_3-δ_^33^, La_0.7_Sr_0.3_VO_3_^34^ and Ba_2_FeMoO_6-δ_^35^, and higher than both La_0.6_Sr_0.4_Fe_0.9_Mn_0.1_O_3_^36^ and PrBaMn_2_O_5+δ_^37^ (Supplementary Table 2).

As a mixed ionic-electronic conducting anode, on the other hand, the anode performance should also strongly depend on oxygen self-diffusion (D*) and surface exchange rate (k*). These two processes could allow the electro-oxidation process to extend from three-phase electrode/electrolyte/gas boundary to the anode surface, then leading to a catalytic enhancement. In this regard, Tarancón *et al.*^38^ suggested that low electrode polarization resistance could be achieved with requirements of k*D* > 10^−14^ cm^3^s^−2^ and k*/D* < 100 cm^−1^. As believed, the special structural feature of layered perovskite may be able to facilitate rapid oxygen mobility and surface exchange in PBFM anode. The anisotropy of oxygen diffusion due to the presence of A-site cation ordered structure with alternating AO_6/2_ and A'O_6/2_ top-corner-shared octahedra may be significantly enhanced, where the rich oxygen vacancies are mainly located in the rare earth planes along the a axis. For example, Taskin *et al.*^22^ investigated the re-oxidation kinetics of GaBaMn_2_O_5+δ_ and GaBaCo_2_O_5+δ_ in which the A cation lattice could be ordered or disordered depending on the synthesis process, and found a remarkable enhancement of oxygen diffusion at rather low temperatures, exceeding 10^−5^ cm^2^/s at 600 °C. Kim *et al.*^21^ applied the isotopic diffusion measurements on PrBaCo_2_O_5+δ_ and reported high values of D*, e.g. > 10^−7^ cm^2^ s^−1^ at 500 °C, despite the porosity of only 90% could give rise to an overestimated value.

Furthermore, the porous thin layer of PBFM (~10 μm) might also facilitate the minimization of polarization resistance. In conventional Ni-YSZ cermet anode, Ni can provide high electrical conductivity for electron transport in the process of reaction and also current collection in the thick substrate. For PBFM anode, the thin layer should be able to relieve the suffering of relatively lower conductivity for the same purposes. Furthermore, the advantage of perovskite anodes also includes the oxygen-rich structure, which could tolerate the loss or gain of lattice oxygen when subjected to change of gas conditions, which might be ascribed to high-coordination BO_6_ octahedra. The rapid recovery of current density when the gas was switched from air to H_2_, as discussed above, indicates that the non-stoichiometry of PBFM should be able to promptly change to original value for recurrence of properties.

In summary, PBFM has therefore been demonstrated as a novel ceramic oxide anode with A-site cation-ordered layered perovskite structure, which shows high electrochemical performance in various fuel conditions. The excellent redox stability and high resistances against both coking and sulfur poisoning are concept-of-proof indicative of that the high catalytic activity for fuel electro-oxidation can be well kept at moderate temperatures in the absence of excess steam. It is also evidently suggested that performance can be further improved by the optimization of microstructure of both electrodes. In addition, this PBFM anode could greatly facilitate its application in anode-supported fuel cells, when some technical strategies, such as introducing some electrically conductive metal-phase materials into anode structure, are required to improve the total anode conductivity and catalytic activity.

## Methods

Layered perovskite oxide of (PrBa)_0.95_(Fe_0.9_Mo_0.1_)_2_O_5+*δ*_(PBFM) was synthesized using modified Pechini process^39^, where citrate and ethylene diamine tetraacetic acid (EDTA) were employed as parallel complexing agents. In the synthesis, Pr_6_O_11_ was first dissolved in nitric acid; the calculated amounts of Ba(NO_3_)_2_, Fe(NO_3_)_3_·9H_2_O and Mo_7_(NH_4_)_6_O_24_∙4H_2_O were dissolved in EDTA-NH_3_ aqueous solution under heating and stirring conditions. An appropriate amount of citric acid was then added into the solution. After converted into a viscous gel under heating and stirring conditions, the solution was ignited to flame and resulted in an ash-like material. Then, this ash-like material was calcined in air at 1000 °C for 10 hours and then in 5% H_2_ at 900 °C for 5 hours to obtain the final material (PBFM). The phase structure of PBFM was analyzed by X-ray powder diffraction by Cu-Kα radiation (D/Max-gA, Japan). A scan rate of 1 ° min^−1^ was used in the range of 20 ° < 2*θ* < 80 °. The anode particles were analyzed by transmission electron microscopy (TEM, JEOL 2100F) operating at 200 kV equipped with energy-dispersive X-ray spectroscopy (EDX) to obtain the details about crystal lattice, element distribution and selected area electron diffraction pattern. XPS was conducted on a Kratos Axis Ultra DLD instrument. Thermogravimetric analysis (Netzsch STA 449) was performed at 25-900 °C with a heating/cooling rate of 2 °C min^−1^ in air or 5% H_2_ to characterize the loss process of lattice oxygen. The rectangular bar of PBFM was cold pressed and consequently sintered at 1300 °C for 10 h to form dense pellet with relative density of 95%. Electrical conductivity of the PBFM was measured as a function of temperature using a direct current four-probe technique (Agilent 34001A) in air and 5% H_2_, respectively. The area-specific-resistance (ASR) values were measured using a symmetric cell PBFM|LSGM|PBFM in dry air, humid H_2_ and CH_4_ (~3% H_2_O) separately, at different temperatures. The full fuel cells with layered PBFM as anode and cobalt-containing layered oxide PrBaCo_2_O_5+δ_ (PBCO) as cathode were prepared based on LSGM electrolyte support. The electrode inks consisting of PBFM (or PBCO) were then applied to the either side of the LSGM electrolyte by brush painting, and then fired at 1000 °C in air for 5 hours to form a porous anode and cathode, respectively. The resulting electrode had a thickness of ∼ 10 μm and an effective area of 0.25 cm^2^.

The button cells were sealed onto a home-made alumina tube using a sliver paste. The cells were tested from 600 to 800 °C with the static air as oxidant and humid hydrogen (~3% H_2_O), methane or H_2_S-containing H_2_ as fuel with a flow rate of 80 ml min^−1^. The voltage-current curves were recorded by DC load at a scanning rate of 50 mV s^−1^. The electrochemical impedance spectra were obtained over a frequency range from 0.01 to 10^5^ Hz under the open-circuit conditions using an electrochemical station (Zennium ZAHNER). A field-emission scanning electron microscope (JSM-6301F) was used to observe the microstructure of the post-test cells.

## Additional Information

**How to cite this article**: Ding, H. *et al.* A High-Performing Sulfur-Tolerant and Redox-Stable Layered Perovskite Anode for Direct Hydrocarbon Solid Oxide Fuel Cells. *Sci. Rep.* **5**, 18129; doi: 10.1038/srep18129 (2015).

## Supplementary Material

Supplementary Information
