# Supplementary material for: A High-Performing Sulfur-Tolerant and Redox-Stable Layered Perovskite Anode for Direct Hydrocarbon Solid Oxide Fuel Cells
Source: Sci Rep. 2015 Dec 9;5:18129. doi: 10.1038/srep18129 (PMC4673446; doi:10.1038/srep18129)
Supplement: Supplementary Information [file srep18129-s1.pdf]

# Supplementary Information

## A High-Performing Sulfur-Tolerant and Redox-Stable Layered Perovskite Anode for Direct Hydrocarbon Solid Oxide Fuel Cells

Hanping Ding[1,3]*, Zetian Tao[2], Shun Liu[1], Jiujun Zhang[4]*

[1]*School of Petroleum Engineering, Xi'an Shiyou University, Xi'an 710065, China*

[2] *Key Laboratory for Advanced Technology in Environmental Protection of Jiangsu Province, Yancheng Institute of College, Yancheng, Jiangsu Province, China*

[3]*Colorado Fuel Cell Center, Department of Mechanical Engineering, Colorado School of Mines, Golden CO 80401, USA*

[4]*Energy, Mining & Environment, National Research Council of Canada, Vancouver, BC V6T 1W5, Canada*

*** Correspondence should be addressed to:** H. P. Ding (hding@mines.edu); J. J. Zhang (Jiujun.zhang@nrc.gc.ca).

**Methods**

In order to measure the electrical conductivity as a function of temperature and thermal expansion coefficient, the rectangular bars (25 mm × 6 mm × 2 mm) of PBFM were sintered at 1300 $^oC$ for 10 hours to make dense bars. The electrical conductivities in air and 5% $H_2$ were measured using four-probe method in the temperature range of 400 to 800 $^oC$. The thermal expansion as a function of temperature was measured by dilatometer (Netzsch DIL 402 PC) in air from room temperature to 900 $^oC$, and the thermal expansion coefficient was obtained by fitting the nearly linear curve. The difference of oxygen losses in air and 5% $H_2$ for PBFM anode was compared by using thermogravimetric analysis (TGA) performed at 25-900 $^oC$ with a

heating/cooling rate of 2 $^{o}C$ min$^{-1}$. The weight loss or gain process was recorded in both heating and cooling processes.

The LSGM powders were uniaxially pressed at 250 MPa to form electrolyte pellets (15 mm in diameter) which were subsequently sintered at 1400 °C in air for 20 hours at a ramping rate of 3 °C min$^{-1}$. The fine PBFM powders were mixed thoroughly with a 6 wt% ethylcellulose-terpineol binder to prepare the anode slurry, which was then painted on either side of LSGM electrolyte, and sintered at 1000 $^{o}C$ for 3 hours in air to form symmetric cells. The area-specific-resistance (ASR) values were determined by impedance spectroscopy in different gas conditions such as dry air, humid $H_2$ and $CH_4$ (~3% $H_2O$), respectively.

The single cells with a structural configuration of PBFM|LSGM|PBCO were measured in various conditions. Firstly, the fuel cell was measured in humid $H_2$ and $CH_4$ from 600 to 800 $^{o}C$, and then discharged at a constant current load of 1.0 A cm$^{-2}$ at 700 $^{o}C$ for 450 hours in $H_2$ and 0.5 A cm$^{-2}$ at 750 $^{o}C$ for 420 hours in $CH_4$. Secondly, it was operated in $H_2$ with different concentrations of $H_2S$ from 0 to 100 ppm at 750 $^{o}C$. When the fuel was fixed at $H_2$ (30 ppm), the I-V curves were recorded from 600 to 800 $^{o}C$. The long-term stability was also investigated under a constant current load of 1.0 A cm$^{-2}$ at 750 $^{o}C$ for 520 hours. Thirdly, at 750 $^{o}C$, the fuel gas was switched between pure $H_2$, $CH_4$ and 30 ppm $H_2S$-containing $H_2$ and the terminal voltage was recorded when the current density was fixed at 0.8 A cm$^{-2}$. Lastly, the redox stability of PBFM anode was performed. By switching the gas between hydrogen and air, the cell voltage was simultaneously monitored at 600 $^{o}C$ while the cell was discharged at 0.35 A cm$^{-2}$. The impedance spectra were measured before and after the redox stability.

The optimization of anode microstructure was performed by ion infiltration of precursor solution prepared by stoichiometric amounts of metal nitrates. The anode frame was fabricated

by painting PBFM slurry onto LSGM electrolyte with a subsequent sintering at 1000 $^oC$ for 3 hours, to form a porous anode layer. The precursor solution, which was prepared by dissolving $Pr(NO_3)_x$ (reaction product from $Pr_6O_{11}$ and $HNO_3$), $Ba(NO_3)_2$, $Fe(NO_3)_3 \cdot 9H_2O$ and $Mo_7(NH_4)_6O_{24}(H_2O)_4$ in distilled water at a molar ratio of Pr : Ba : Fe : Mo = 0.95 : 0.95 : 1.8 : 0.2, was impregnated into the anode frame by fast vacuum and then fired at 800 $^oC$ for 15 minutes with a heating and cooling rate of 10 $^oC$ $min^{-1}$. The impregnated nanoparticles were finally in-situ reduced when it was tested at 800 $^oC$ in $H_2$.

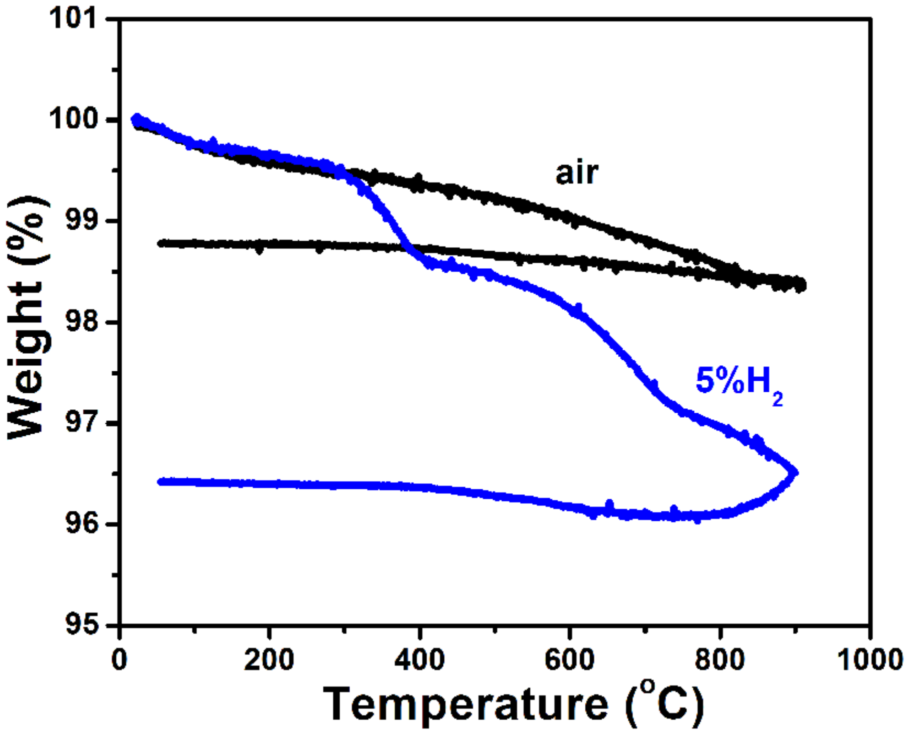


**Figure S1.** TGA of layered PBFM is carried out in both air and 5%$H_2$ to evaluate the loss of lattice oxygen content. Upon heating in air, the weight loss below 350 $^oC$ is due to desorption of water and then the lattice oxygen starts to loss with increasing temperature. At 900 $^oC$, PBFM shows a total 1.5% decrease in weight compared to its initial weight. In 5% $H_2$, PBFM exhibits the first sharp weight loss around 300-400 $^oC$ and the second rapid loss around 600-700 $^oC$, respectively, which can be mostly ascribed to the formation of oxygen vacancies. The total weight loss in 5%$H_2$ is 3.5%. For each case, PBFM gained slight weight during cooling process, which corresponds to the re-oxidation of the material.

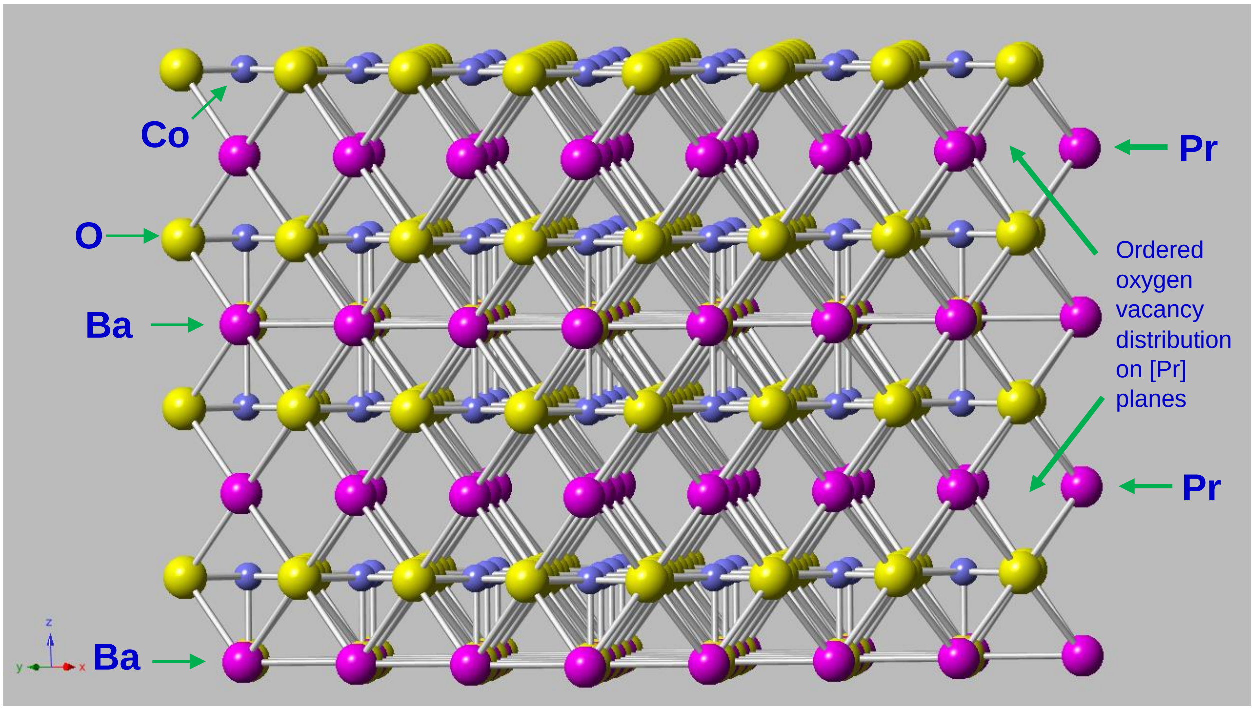


**Figure S2.** Schematic of crystal structure of layered perovskite PBFM oxide. A significant difference of ionic size between Pr and Ba cations results in the alternative distribution of [$PrO_x$] and [BaO] layers along [001] axis. The oxygen vacancies are mostly localized in the Pr planes, resulting in disorder-free channels for mobility of oxygen ions.

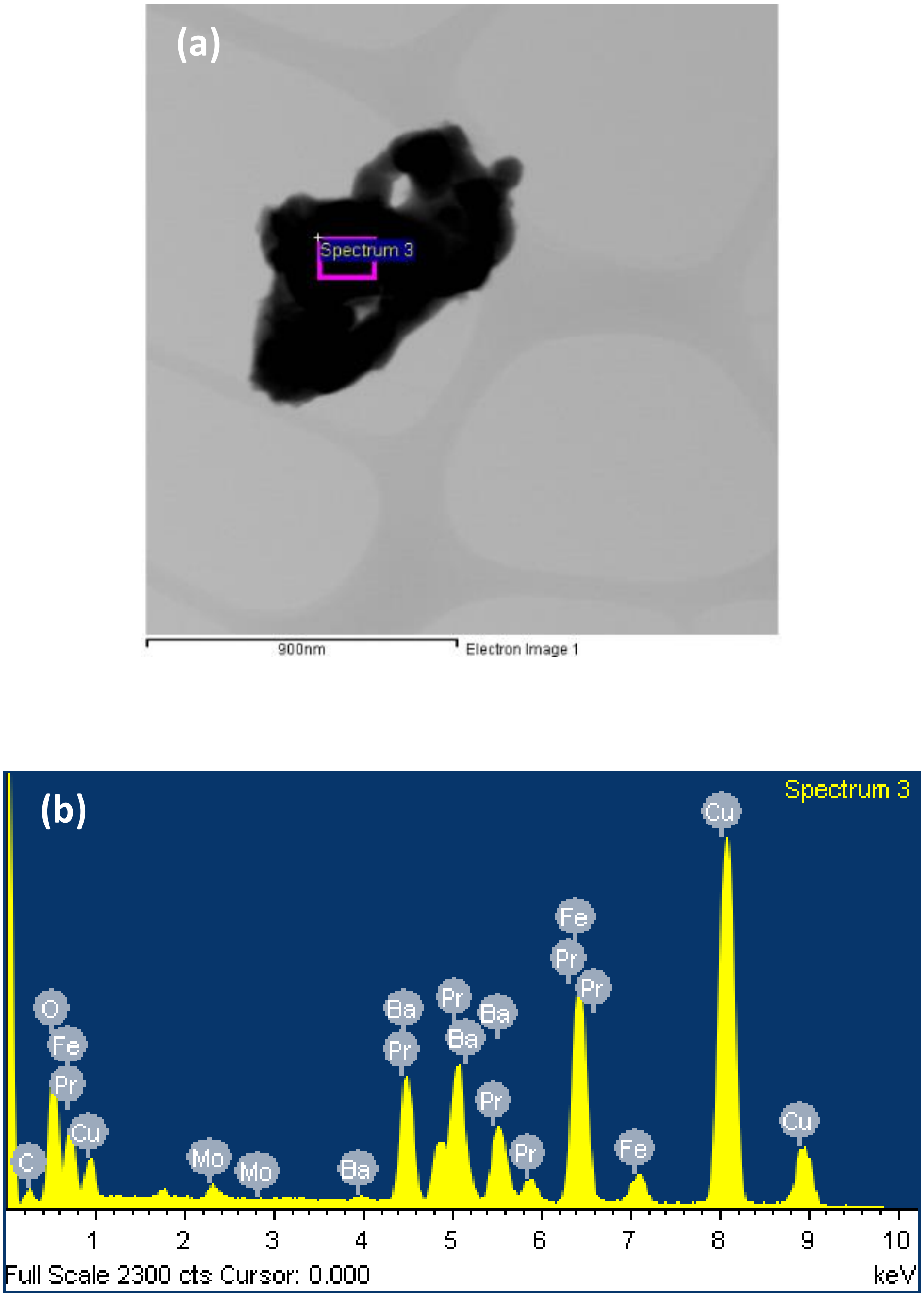


**Figure S3.** (a) Morphology of a layered-perovskite PBFM particle by TEM; (b) analysis of element composition on a selected area (box in a) by energy-dispersive X-ray spectroscopy equipped in TEM instrument.

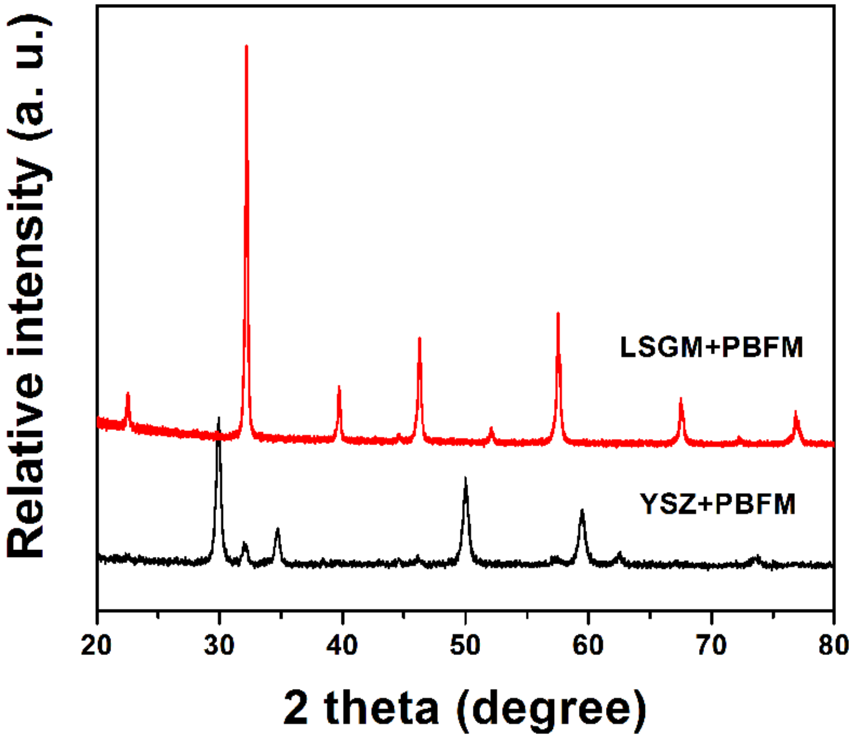


**Figure S4.** Chemical compatibility between PBFM anode and LSGM (or YSZ) electrolytes. The mixed powders (50 : 50 wt%) is fired at 1000 $^{o}$C for 10 hours in air.

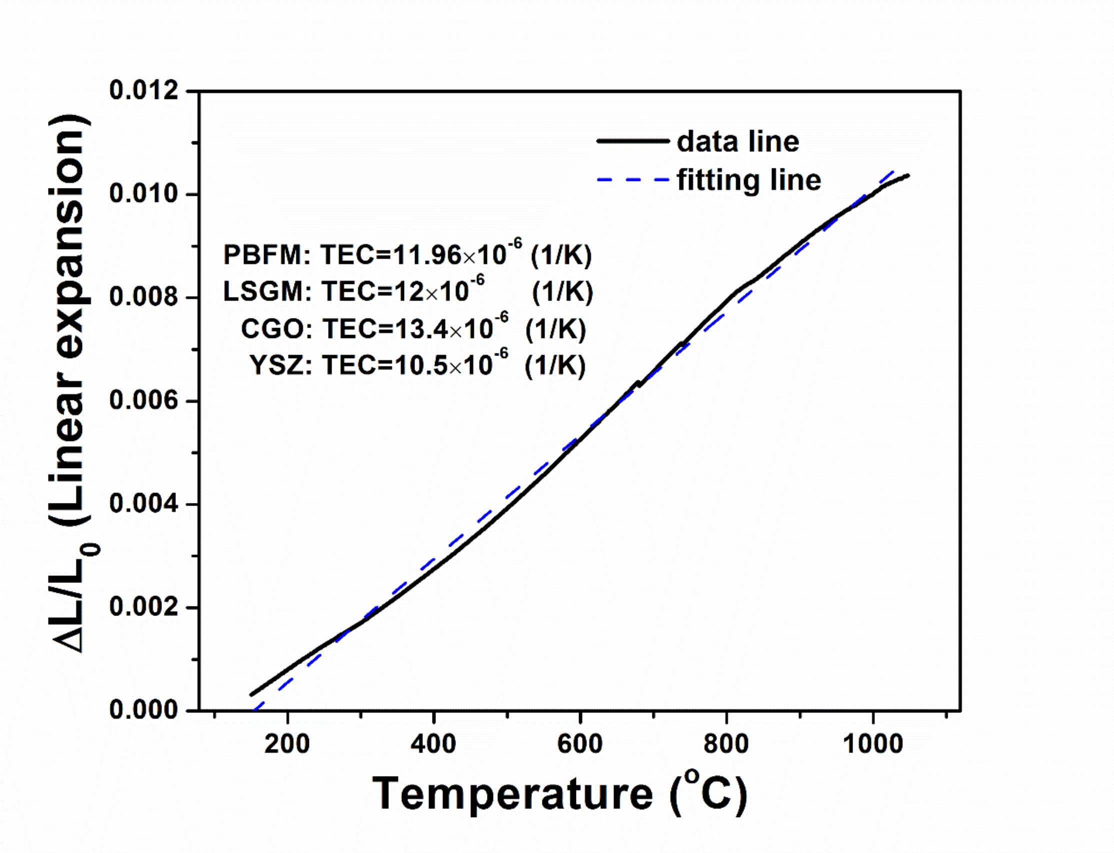


**Figure S5.** Thermal expansion coefficients (TECs) of PBFM perovskite and some electrolyte materials of LSGM, YSZ ($Y_{0.08}Zr_{0.92}O_2$) and CGO ($Gd_{0.1}Ce_{0.9}O_{1.9}$).

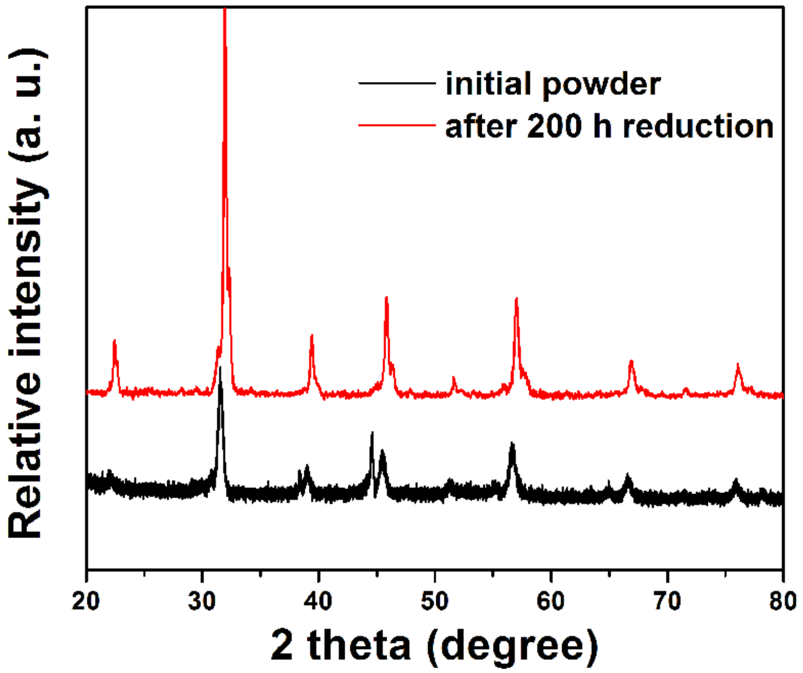


**Figure S6.** Long-term chemical stability of PBFM anode in 5% $H_2$ at 1000 $^oC$ for 200 hours.

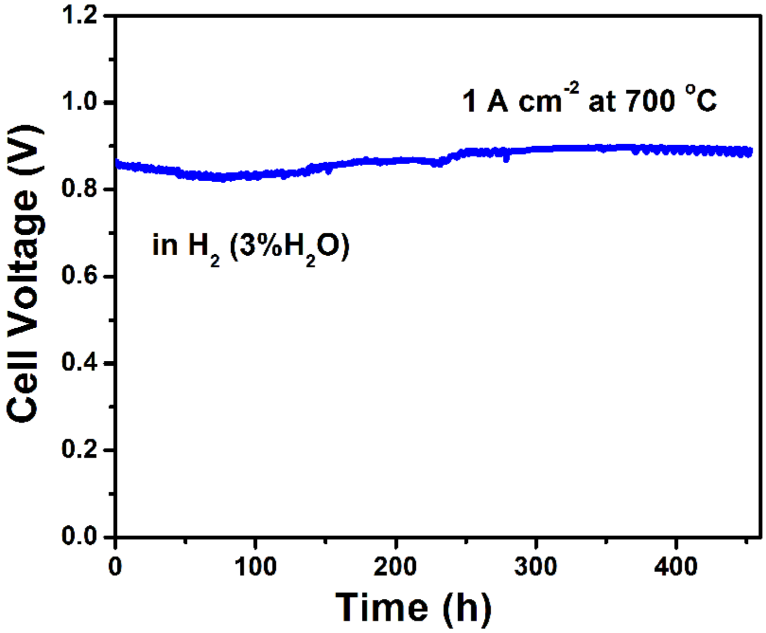


**Figure S7.** Long-term stability for PBFM anode under a constant current load of 1.0 A cm$^{-2}$ at 700 $^{o}$C in $H_2$.

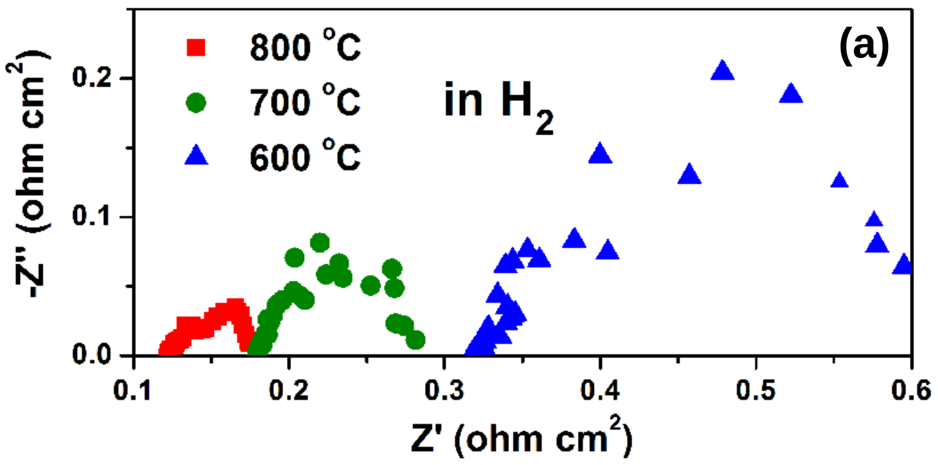


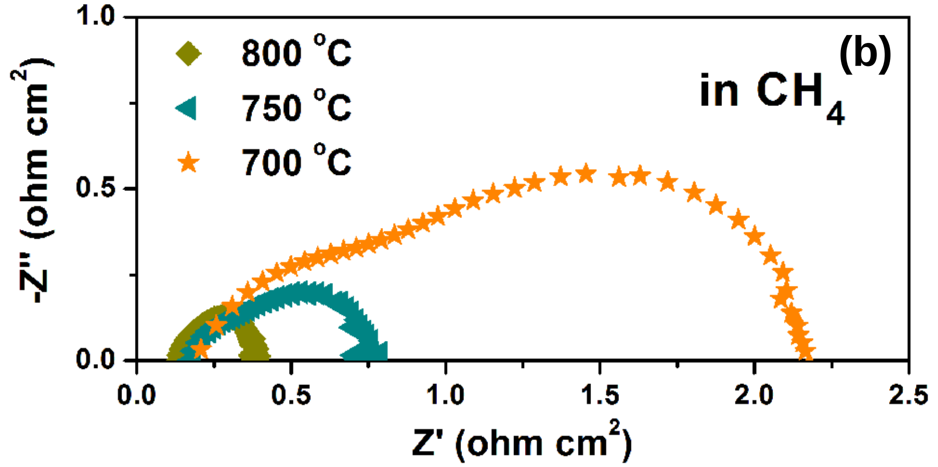


**Figure S8.** Impedance spectra of layered PBFM anode in various fuels: (a) humid $H_2$ (~3% $H_2O$) and (b) humid $CH_4$ (~3% $H_2O$), operating at different temperatures.

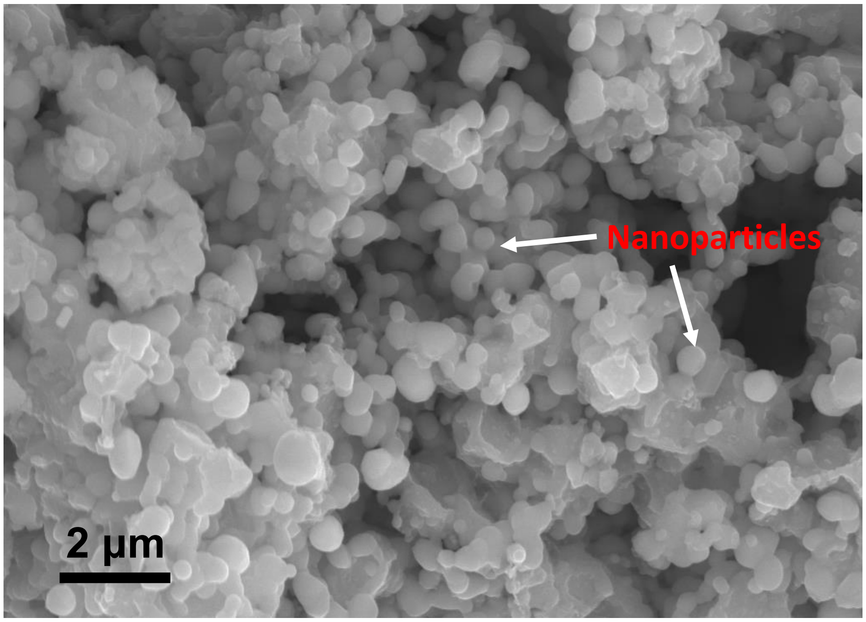


**Figure S9.** Microstructure of PBFM anode layer uniformly infiltrated with PBFM nanoparticles.

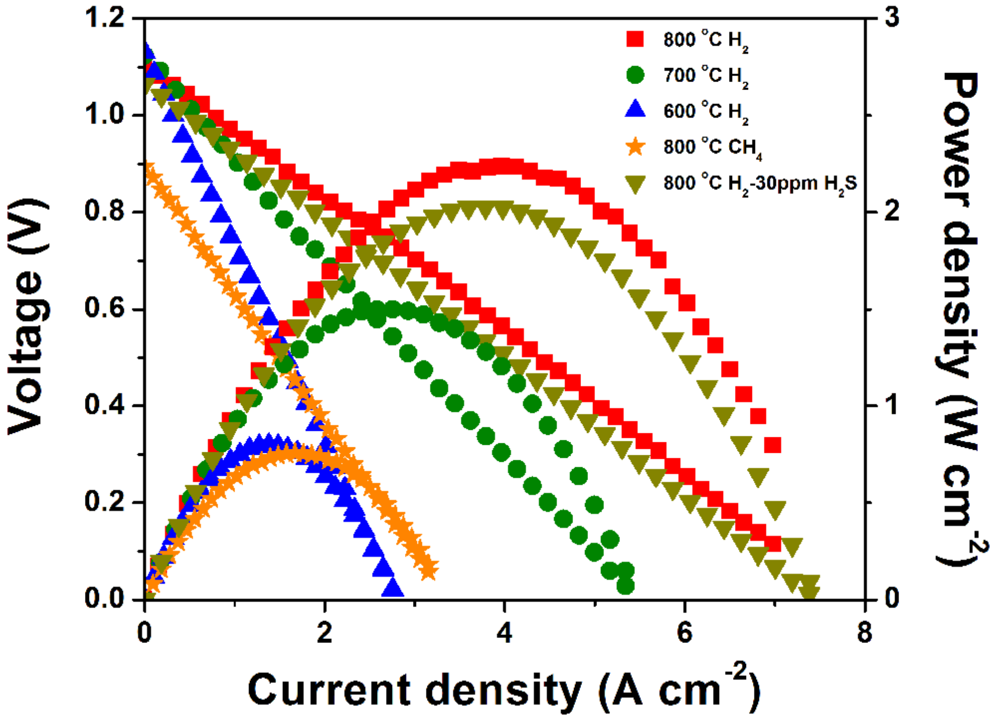


**Figure S10.** I-V and I-P curves of PBFM anode with active catalyst of PBFM nanoparticles, operating in different fuels.

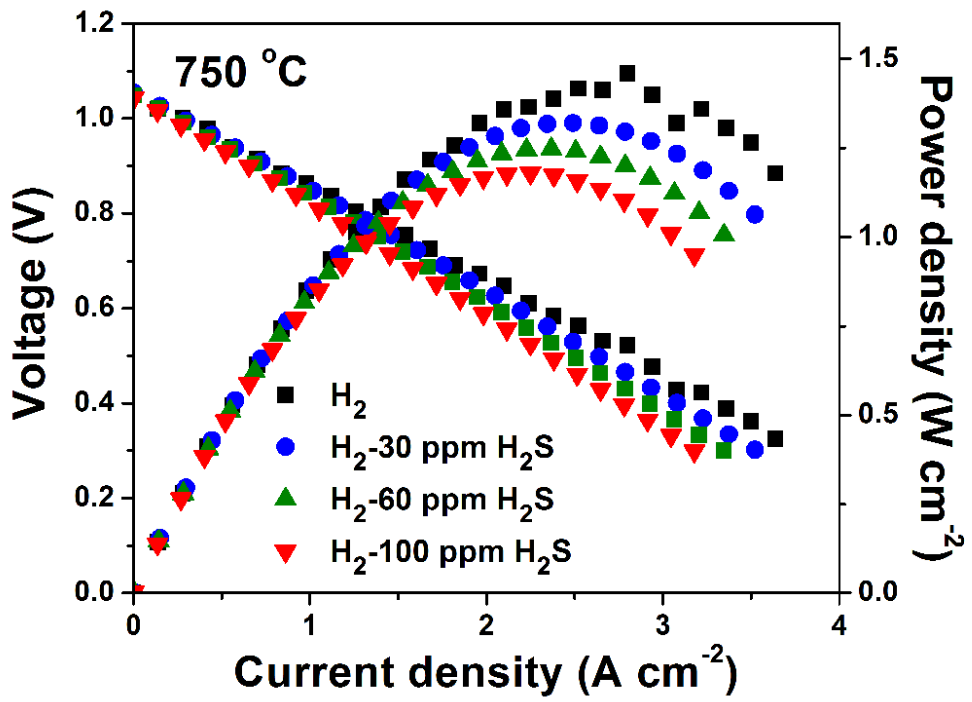


**Figure S11.** I-V and I-P curves of PBFM anode at 750 °C in $H_2$ with different concentrations of $H_2S$ from 0 to 100 ppm.

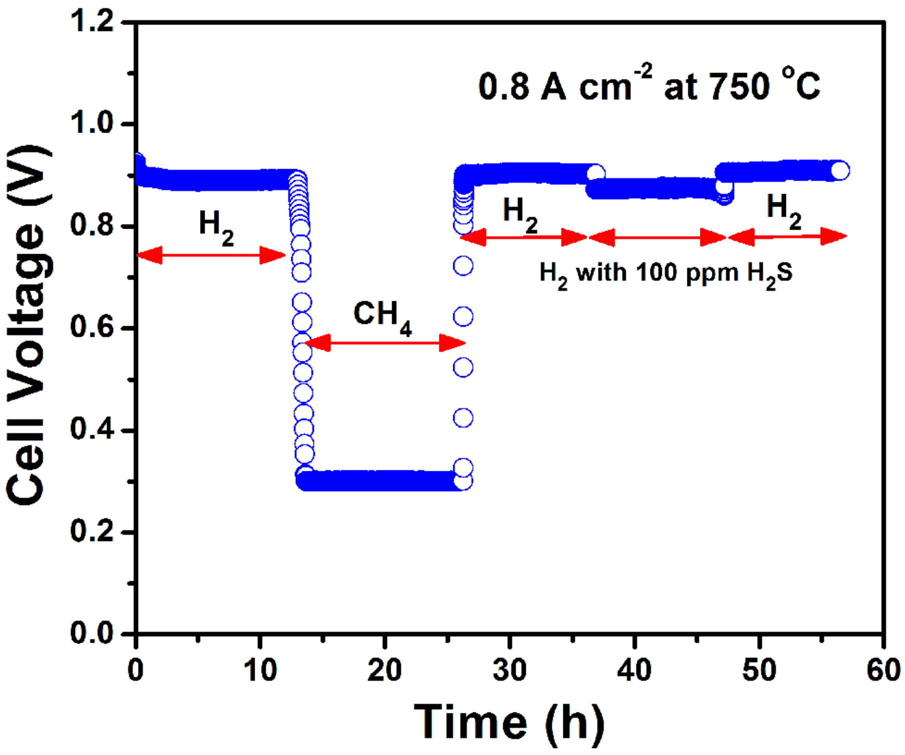


**Figure S12.** Observation of terminal voltage under a constant current load of 0.8 A $cm^{-2}$ at 750 °C when the fuel is switched among different gases: $H_2$, $CH_4$, $H_2$-100 ppm $H_2S$.

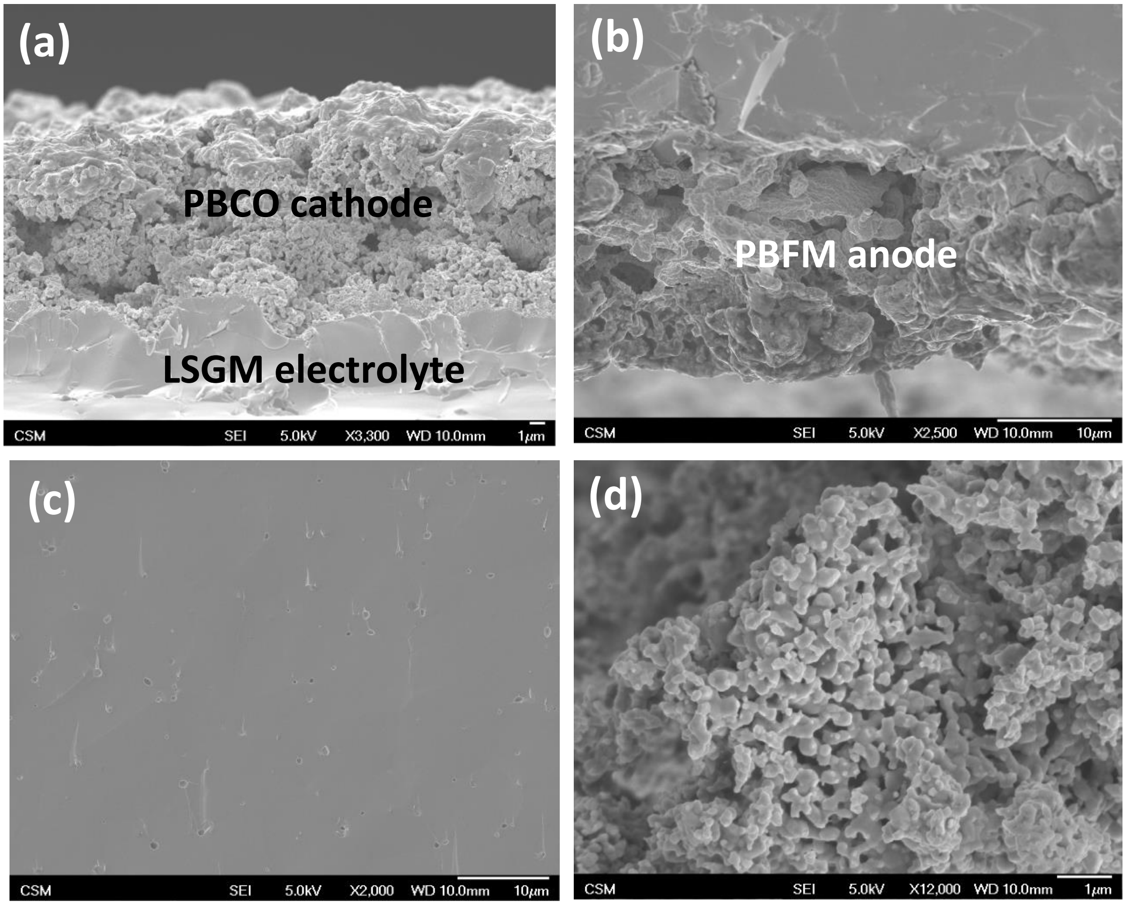


**Figure S13.** Microstructure of LSGM electrolyte-supported fuel cell with PBFM anode after various measurements. (a) Electrolyte/PBCO cathode interface; (b) Electrolyte/PBFM anode interface; (c) Dense LSGM electrolyte; (d) Morphology of PBFM anode layer.

**Table S1.** Fuel cell performance of selected oxide anode materials.

| Anode composition | Electrolyte (thickness: µm)/cathode | Temp. (ºC) | Fuel | $P_{max}$ (W cm$^{-2}$) | Ref. |
|---|---|---|---|---|---|
| $La_{0.4}Sr_{0.6}TiO_3$ | YSZ (160 µm)/LSM | 1000 | $H_2$ | 0.26 | [1] |
| $La_{0.2}Sr_{0.8}Ti_{0.98}Co_{0.02}O_3$ + GDC + Ni | LSGM (250 µm)/ $La_{0.6}Sr_{0.4}Co_{0.2}Fe_{0.8}O_{3-\delta}$ | 800 | $H_2$ | 0.66 | [2] |
| $La_{0.7}Sr_{0.3}VO_3$ | YSZ (100 µm)/$La_{0.85}Sr_{0.15}MnO_3$ | 950 | $H_2$ | 0.16 | [3] |
| $La_{0.75}Sr_{0.25}Cr_{0.5}Mn_{0.5}O_{3-\delta}$ | YSZ (300 µm)/$La_{0.8}Sr_{0.2}MnO_3$ | 900 | $H_2$ | 0.5 | [4] |
| $Sr_2Fe_{1.5}Mo_{0.5}O_6$ | LSGM (265 µm)/ $Sr_2Fe_{1.5}Mo_{0.5}O_6$ | 900 | $H_2$ | 0.84 | [5] |
| | | 900 | $CH_4$ | 0.23 | |
| $Pr_{0.8}Sr_{1.2}(Co,Fe)_{0.8}Nb_{0.2}O_{4+\delta}$ | LSGM (300 µm)/ $Pr_{0.8}Sr_{1.2}(Co,Fe)_{0.8}Nb_{0.2}O_{4+\delta}$ | 850 | $H_2$ | 0.96 | [6] |
| | | 850 | $CH_4$ | 0.6 | |
| $La_{0.6}Sr_{0.4}Fe_{0.9}Mn_{0.1}O_3$ + $Ce_{0.6}Mn_{0.3}Fe_{0.1}O_2$ | LSGM (~300 µm)/$Sm_{0.5}Sr_{0.5}CoO_3$ | 800 | $H_2$ | 0.32 | [7] |
| | | 800 | $CH_4$ | 0.24 | |

**Table S2.** Electrical conductivities of typical oxide anode materials in $H_2$.

| Anode composition | Electrical conductivity (S cm$^{-1}$) | Temperature (ºC) | Gas condition | Ref. |
|---|---|---|---|---|
| $La_{0.3}Sr_{0.7}TiO_{3-\delta}$ | ~100 | 1000 | 5% $H_2$ | [8] |
| $SrTi_{0.92}Nb_{0.08}O_{3-\delta}$ | ~10 | 800 | $H_2$ | [9] |
| $La_{0.7}Sr_{0.3}VO_3$ | ~150 | 800 | $H_2$ | [10] |
| $Ba_2FeMoO_{6-\delta}$ | ~100 | 800 | $H_2$ | [11] |
| $Sr_2Fe_{1.5}Mo_{0.5}O_6$ | 310 | 780 | $H_2$ | [5] |
| $La_{0.6}Sr_{0.4}Fe_{0.9}Mn_{0.1}O_3$ | ~20 | 800 | $H_2$ | [7] |
| $Ce_{0.6}Mn_{0.3}Fe_{0.1}O_2$ | ~10 | 800 | $H_2$ | [7] |
| $PrBaMn_2O_{5+\delta}$ | 8 | 800 | 5% $H_2$ | [12] |

## References

1. Marina, O. A., Canfield, N. L. & Stevenson, J. W. Thermal, electrical, and electrocatalytical properties of lanthanum-doped strontium titanate. *Solid State Ionics* **149**, 21-28 (2002).

2. Yoo, K. B., Park, B. H. & Choi, G. M. Stability and performance of SOFC with $SrTiO_3$-based anode in $CH_4$ fuel. *Solid State Ionics* **225**, 104-107 (2012).

3. Cheng, Z., Zha, S. W., Aguilar, L., Wang, D., Winnick, J. & Liu, M. L. A solid oxide fuel cell running on $H_2S/CH_4$ fuel mixtures. *Electrochemical and Solid-State Letters* **9**, A31-A33 (2006).

4. Tao, S. W. & Irvine, J. T. S. A redox-stable efficient anode for solid-oxide fuel cells, *Nat. Mater.* **2**, 320-323 (2003).

5. Liu, Q., Dong, X. H., Xiao, G. L., Zhao, F. & Chen, F. L. A novel electrode material for symmetrical SOFCs. *Adv. Mater.* **22**, 5478-5482 (2010).

6. Yang, C. H., Yang, Z. B., Jin, C., Xiao, G. L., Chen, F. L. & Han, M. F. Sulfur-tolerant redox-reversible anode material for direct hydrocarbon solid oxide fuel cells. *Adv. Mater.* **24**, 1439-1443 (2012).

7. Shin, T. H., Ida, S. & Ishihara, T. Doped $CeO_2$-$LaFeO_3$ composite oxide as an active anode for direct hydrocarbon-type solid oxide fuel cells. *J. Am. Chem. Soc.* **133**, 19399-19407 (2011).

8. Hashimoto, S., Kindermann, L., Larsen, P. H., Poulsen, F. W. & Mogensen M. Conductivity and expansion at high temperature in $Sr_{0.7}La_{0.3}TiO_{3-\alpha}$ prepared under reducing atmosphere, *J. Electroceram.* **16**, 103-107 (2006).

9. Karczewski, J., Riegel, B., Gazda, M., Jasinski, P. & Kusz, B. Electrical and structural properties of Nb-doped $SrTiO_3$ ceramics. *J. Electroceram.* **24**, 326-330 (2010).

10. Cheng, Z., Zha, S. W., Aguilar, L. & Liu, M. L. Chemical, electrical, and thermal properties of strontium doped lanthanum vanadate. *Solid State Ionics* **176**, 1921-1928 (2005).

11. Zhang, Q., Wei, T. & Huang, Y. H. Electrochemical performance of double-perovskite $Ba_2MMoO_6$ (M = Fe, Co, Mn, Ni) anode materials for solid oxide fuel cells. *J. Power Sources* **198**, 59-65 (2012).

12. Sengodan, S., Choi, S., Jun, A., Shin, T. H., Ju, Y-W., Jeong, H. Y., Shin, J., Irvine, J. T. S. & Kim, G. Layered oxygen-deficient double perovskite as an efficient and stable anode for direct hydrocarbon solid oxide fuel cells. *Nat. Mater.* **14**, 205-209 (2015).
